# Spectrum of Diagnoses in Female Patients With Proctologic Symptoms Presenting to the Surgery Unit of a Tertiary Care Center

**DOI:** 10.7759/cureus.57600

**Published:** 2024-04-04

**Authors:** Sana Sahar, Tamjeed Gul, Muhammad Ihtesham Khan

**Affiliations:** 1 Department of Surgery, Khyber Teaching Hospital, Peshawar, PAK; 2 Department of Surgery, Mardan Medical Complex, Peshawar, PAK; 3 Department of Pathology, Khyber Medical College, Peshawar, PAK

**Keywords:** sigmoidoscopy, proctoscopy, hemorrhoids, bleeding per rectum, anal fissures

## Abstract

Introduction

Anorectal diseases are prevalent in the general population and may vary from benign disorders to malignant lesions that can metastasize. There is a variety of proctologic symptoms associated with each disease. The incidence of proctologic disease varies in different cultures due to dietary habits and variations in lifestyle. The present study was conducted to determine the spectrum of different proctologic diseases in female patients presenting with proctologic symptoms.

Methods

This cross-sectional study was conducted in the Surgery Department of Mardan Medical Complex, Mardan, and Khyber Teaching Hospital, Peshawar, from January 2022 to January 2023. Female patients with proctologic symptoms were included, while non-consenting patients were excluded. After obtaining a detailed history and examination by the experienced surgeon, digital rectal examination and proctoscopy/sigmoidoscopy were performed where necessary. Diagnoses were made, and the data regarding proctologic symptoms and their corresponding diagnoses was analyzed using Statistical Package for the Social Sciences (SPSS) version 20.0 (IBM SPSS Statistics, Armonk, NY) using mean and standard deviation for quantitative variables and frequency and percentage for qualitative variables.

Results

The mean age of 500 female study participants was 38.35±16.305 (range: 7-108) years. Bleeding per rectum, constipation, and pain per rectum were the commonest proctologic symptoms seen in 341 (68.2%), 287 (57.4%), and 272 (54.4%) cases, respectively. Anal fissures and hemorrhoids were the commonest proctologic diseases seen in 264 (52.8%) and 60 (12%) cases, respectively.

Conclusion

Bleeding per rectum is the commonest proctologic symptom in patients. Anal fissures and hemorrhoids are the commonest proctologic diseases in our setup. Bleeding per rectum and hemorrhoids in the female population cause loss of blood, which in turn will aggravate the clinical picture of underlying anemia, if any.

## Introduction

Proctologic symptoms arise as a result of a wide range of diseases in the anorectal region. These may range from benign disorders of the anorectal region to malignant diseases that can metastasize to distant organs and tissues [1,2]. The diseases of the anorectal region present as proctologic symptoms such as bleeding in stool, painful defecation, and pain in the anal area [1].

Anorectal diseases are common worldwide. The commonest anorectal diseases presenting with proctologic symptoms are hemorrhoids, anal fissures, fecal incontinence, and anorectal abscess. In the United States, the prevalence of hemorrhoids is about 6%, and only one-third of these cases take clinical consultation [3]. The prevalence of hemorrhoids in the United Kingdom is much higher, i.e., 13%-35% [3]. The worldwide incidence of hemorrhoids is 4% [4].

To make a diagnosis of proctologic symptoms, a detailed clinical history is taken from the patient, and a digital rectal examination is performed [5]. In case of suspicion of malignancy, proctoscopy or sigmoidoscopy is performed to inspect the mucosal surface of the anorectal area. In case of mass, a specimen is taken and sent for histopathological evaluation. It is reported that 20% of cases presenting with proctologic symptoms have disease that needs surgical intervention [5].

Anorectal diseases are associated with significant morbidity. Unluckily, patients avoid reporting the symptoms, and the disease worsens with time [6,7]. With time, the underlying disease becomes chronic, and patients get complications [6]. This worsens the quality of life of patients. In complicated cases, making a diagnosis becomes a challenge [7].

To our knowledge, there is no study that determines the common proctologic diseases in the female population in this part of the world where social stigmatization and scarce healthcare facilities are a problem. The current study is conducted to determine common diagnoses of proctologic symptoms in female patients of the districts of Khyber Pakhtunkhwa.

## Materials and methods

This observational cross-sectional study was conducted in the Department of Surgery at Mardan Medical Complex, Mardan, and Khyber Teaching Hospital, Peshawar, after obtaining approval from the institutional ethical review board. The study was conducted from January 2022 to January 2023, i.e., one-year duration. Female patients of all ages presenting with proctologic symptoms such as pain, bleeding, swelling, constipation, prolapse, itching, mucous discharge, and tenesmus were included in the study. Non-consenting patients were excluded from the study. Informed consent was obtained from the patients, and they were explained that their data will be kept confidential. A detailed history was taken from the patients. Examination was done by an experienced surgeon, and findings were noted on a proformas. Digital rectal examination and proctoscopy/sigmoidoscopy were performed where necessary. A biopsy specimen was taken from the gut and sent for confirmation of diagnoses by an experienced histopathologist where necessary. Blood samples were taken and sent for complete blood counts in cases suspicious of anemia and thyroid function tests in cases suspicious of hypothyroidism. Diagnoses were made, and data were analyzed using Statistical Package for the Social Sciences (SPSS) version 20.0 (IBM SPSS Statistics, Armonk, NY) using mean and standard deviation for quantitative variables and frequency and percentage for qualitative variables.

## Results

**Figure 1:**
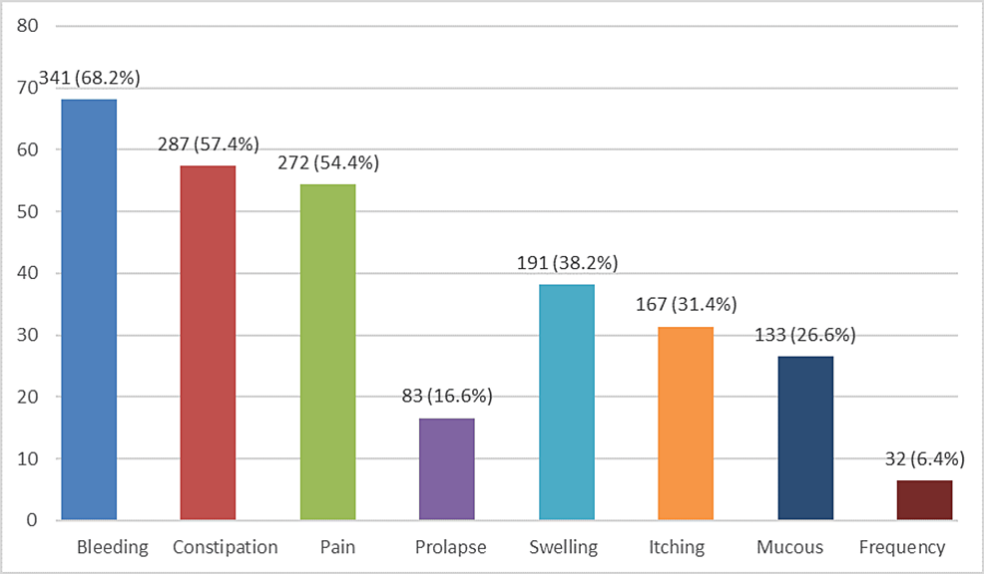
Proctologic symptoms in the study participants Data is presented as frequency (number) and percentage (%).

**Figure 2:**
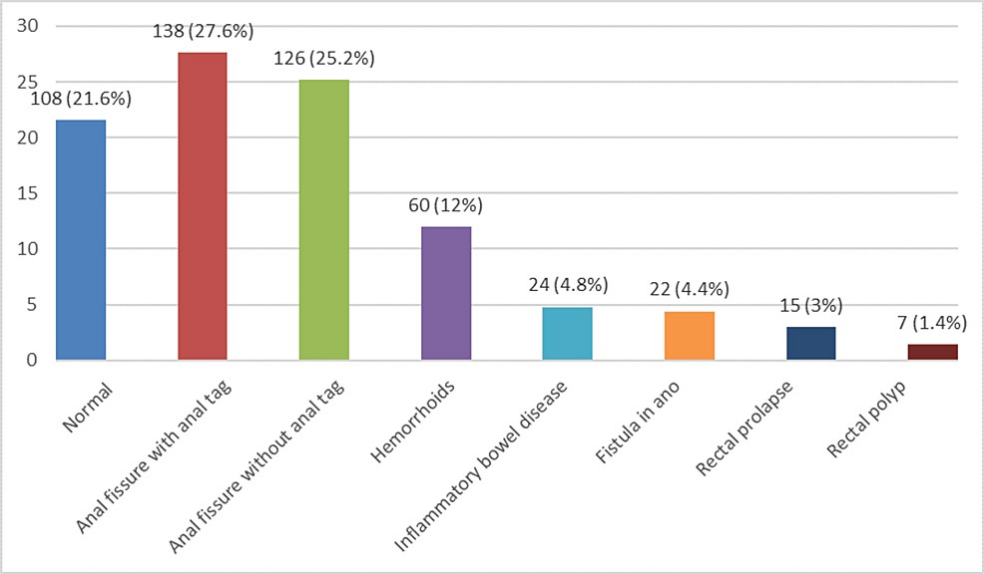
Diagnoses for proctologic symptoms in the study participants (N=500) Data is depicted as frequency (number) and percentages (%).

The study was done on 500 female patients who complained of recurrent proctologic symptoms and did not receive any intervention previously. The mean age of the study sample was 38.35±16.305 years with an age range of 7-108 years. The proctologic symptoms and their diagnoses are depicted in Figure 1 and Figure 2, respectively. The diagnoses with respect to proctologic symptoms are shown in Table 1.

**Table 1:** Diagnoses with respect to proctologic symptoms in the study participants (N=500) Data is shown as frequency (number) and percentage(%). IBD: inflammatory bowel disease

Proctologic symptoms	Bleeding (N=500)		Constipation (N=500)		Pain (N=500)		Prolapse (N=500)		Swelling (N=500)		Itching (N=500)		Mucous (N=500)		Frequency of stool (N=500)	
	Yes (number (%))	No (number (%))	Yes (number (%))	No (number (%))	Yes (number (%))	No (number (%))	Yes (number (%))	No (number (%))	Yes (number (%))	No (number (%))	Yes (number (%))	No (number (%))	Yes (number (%))	No (number (%))	Yes (number (%))	No (number (%))
Diagnosis																
Anal fissure with tag	127 (25.4%)	11 (2.2%)	114 (22.8%)	24 (4.8%)	135 (27%)	3 (0.6%)	2 (0.4%)	136 (27.2%)	138 (27.6%)	0 (0%)	54 (10.8%)	84 (16.8%)	42 (8.4%)	96 (19.2%)	1 (0.2%)	137 (27.4%)
Anal fissure without tag	117 (23.4%)	9 (1.8%)	91 (18.2%)	35 (7%)	124 (24.8%)	2 (0.4%)	3 (0.6%)	123 (24.6%)	1 (0.2%)	125 (25%)	26 (5.2%)	100 (20%)	24 (4.8%)	102 (20.4%)	0 (0%)	126 (25.2%)
Hemorrhoid	60 (12%)	0 (0%)	44 (8.8%)	16 (3.2%)	5 (1%)	55 (11%)	56 (11.2%)	4 (0.8%)	17 (3.4%)	43 (8.6%)	21 (4.2%)	39 (7.8%)	20 (4%)	40 (8%)	0 (0%)	60 (12%)
IBD	22 (4.4%)	2 (0.4%)	1 (0.2%)	23 (4.6%)	1 (0.2%)	23 (4.6%)	0 (0%)	24 (4.8%)	0 (0%)	24 (4.8%)	4 (0.8%)	20 (4%)	3 (0.6%)	21 (4.2%)	23 (4.6%)	1 (0.2%)
Fistula in ano	0 (0%)	22 (4.4%)	4 (0.8%)	18 (3.6%)	1 (0.2%)	21 (4.2%)	0 (0%)	22 (4.4%)	0 (0%)	22 (4.4%)	19 (3.8%)	3 (0.6%)	22 (4.4%)	0 (0%)	0 (0%)	22 (4.4%)
Polyp	7 (1.4%)	0 (0%)	0 (0%)	7 (1.4%)	1 (0.2%)	6 (1.2%)	7 (1.4%)	0 (0%)	1 (0.2%)	6 (1.2%)	1 (0.2%)	6 (1.2%)	1 (0.2%)	6 (1.2%)	0 (0%)	7 (1.4%)
Rectal prolapse	2 (0.4%)	13 (2.6%)	0 (0%)	15 (3%)	2 (0.4%)	13 (2.6%)	15 (3%)	0 (0%)	0 (0%)	15 (3%)	3 (0.6%)	12 (2.4%)	4 (0.8%)	11 (2.2%)	1 (0.2%)	14 (2.8%)
Normal	6 (1.2%)	102 (20.4%)	33 (6.6%)	75 (15%)	3 (0.6%)	105 (21%)	0 (0%)	108 (21.6%)	34 (6.8%)	74 (14.8%)	19 (3.8%)	89 (17.8%)	17 (3.4%)	91 (18.2%)	7 (1.4%)	101 (20.2%)

Figure 1 shows that out of 500 patients, 341 (68.2%) cases presented with bleeding per rectum, while constipation and pain per rectum were seen in 287 (57.4%) and 272 (54.4%) cases, respectively.

Figure 2 shows that out of 500 patients, 138 (27.6%) cases had a diagnosis of anal fissure with anal tags, while 126 (25.2%) patients presented with anal fissure without tags. Thus, the total number of patients presenting with anal fissures turned out to be 264 (52.8%), which constituted more than half of the patients. The next common diagnosis was hemorrhoids, which was seen in 60 (12%) cases.

Table 1 shows that the commonest symptoms in patients with anal fissure with tag were swelling, pain, and bleeding, which were seen in 138 (27.6%), 135 (27%), and 127 (25.4%) cases, respectively. Rectal prolapse was the commonest symptom of hemorrhoids, which was seen in 56 (11.2%) patients with hemorrhoids. Frequency of stool was the commonest symptom in inflammatory bowel disease, which was seen in 23 (4.6%) patients with inflammatory bowel disease.

## Discussion

Anorectal diseases are common and affect about one-fourth of the general population [1]. Due to the increasing number of complicated cases related to proctologic diseases, there is currently an emerging field of colorectal surgery to meet the needs of the general population [8]. Unluckily, the incidence of proctologic disorders in females is not appropriately reported due to lack of research and lack of patient referral.

Anorectal diseases worsen the quality of life of patients. Patients are reluctant to consult surgeons, and the disease keeps lingering [6,7]. According to a report, about 80% of cases with proctologic symptoms do not consult their consultant [5]. This is commonly seen in conservative societies where anorectal diseases are considered a stigma and therefore kept a secret. This is true, especially in females. Delays in diagnosis and treatment cause the disease to become chronic and develop complications [6]. Diagnosing such complicated cases can be a diagnostic challenge prompting the adoption of a multidisciplinary approach involving a colorectal specialist, urologist, and gastroenterologist [7].

In the current study, it was observed that the patients were of middle age. Similar data was reported by Oumar et al. [9]. However, Mariko et al. [10] reported a comparatively younger age female population in their study [10].

In the current study, the commonest proctologic symptoms were bleeding, constipation, and pain. Abramowitz et al. [6] reported similar findings from France. In our study, when different diagnoses were considered, it was seen that anal fissures and hemorrhoids were the commonest diagnoses in the female population. A similar pattern of diagnosis was reported by Abramowitz et al. [6] from France and Yadav et al. [1] from Nepal. Mariko et al. [10] from Mali also reported anal fissures and hemorrhoids as the commonest entities in their population [10]. Similar data is reported from Central Africa, Dakar, and India [11-13]. Perveen et al. [5] from Karachi reported hemorrhoids as the commonest anorectal disease.

An anal fissure is characterized by superficial injury or tear in the mucocutaneous junction of the anal canal [9]. Constipation is the risk factor for the development of anal fissures. The disease presents with severe pain during defecation. Due to straining, there appear tears in the fissure that cause the presence of blood in the stool [9]. According to research, the lifetime risk of having anal fissure in any individual is 7% [9]. The condition is common in the female population, especially in pregnant females [9].

Hemorrhoid is another anorectal disease characterized by abnormal dilatation of submucous veins in the anal canal that bleed on straining while defecating. In chronic untreated hemorrhoids, the bleeding can be so profuse that patients develop anemia. Recently, it has been reported that untreated hemorrhoids are associated with a higher risk of colorectal adenomas [14].

The limitation of the current study was that the study was conducted in only two tertiary care centers. Therefore, the result may not represent the general population. We recommend that bigger studies should be done including patients from multiple healthcare centers so that bigger data is generated that can truly represent the whole population.

## Conclusions

Bleeding per rectum is the commonest proctologic symptom in patients. Anal fissures and hemorrhoids are the commonest proctologic diseases in females in the province of Khyber Pakhtunkhwa and should be considered in the differential diagnosis of patients presenting with proctologic symptoms. Bleeding per rectum and hemorrhoids in the female population cause loss of blood, which in turn will aggravate the clinical picture of underlying anemia, and the disease will present with complications, thus adding to disease morbidity. Early referral for prompt diagnosis and treatment of these disease entities is recommended to prevent disease complications.
